# The Conducting Tissues of the Mouse Heart

**DOI:** 10.3390/jcdd12110452

**Published:** 2025-11-20

**Authors:** Yolanda Macías, Damián Sánchez-Quintana, Eduardo Back Sternick, Robert H. Anderson

**Affiliations:** 1 Department of Medical and Surgical Therapeutics, Centre of Plasencia, University of Extremadura, 10600 Cáceres, Spain; yolmacgan@gmail.com; 2 Department of Human Anatomy and Cell Biology, Faculty of Medicine and Health Sciences, University of Extremadura, 06006 Badajoz, Spain; 3 Arrhythmia and Electrophysiology Department, Biocor Institute, Nova Lima 34000-000, Brazil; eduardosternick@gmail.com; 4 Biosciences Division, Newcastle University, Newcastle-upon-Tyne NE1 7RU, UK; sejjran@ucl.ac.uk

**Keywords:** sinus node, atrioventricular node, atrioventricular conduction axis, internodal atrial myocardium, ventricular bundle branches

## Abstract

**Background:** Since the study of Lev and Thaemert in 1973, little has been published concerning the overall arrangement of the murine conduction tissues, in particular with relation to gross anatomical landmarks. We recently emphasised the potential value of the mouse as an experimental animal in determining the role, if any, of the superior septal pathways in ventricular activation, comparing the findings to the arrangement in the human heart. Our previous study, however, was confined to the atrioventricular conduction axis. In the light of recent controversies regarding atrial conduction, we have now extended our study to evaluate the overall arrangement of the conduction tissues in the murine heart. **Methods:** We have re-examined serially sectioned histological datasets previously prepared from adult mouse hearts, incorporating new datasets to permit assessment in the three orthogonal planes, correlating the histological findings with the gross anatomy as revealed in episcopic datasets prepared from late foetal and neonatal mice. **Results:** The overall arrangement of the conduction tissues is comparable with the human arrangement, but with subtle differences. The sinus node straddles the superior cavoatrial junction, rather than being embedded within the terminal groove. Conduction from the node to the atrial walls is through working atrial myocardium. The atrioventricular conduction axis, as we have recently emphasised, is much more comparable, in particular with presence of well-formed nodo-ventricular and fasciculo-ventricular pathways. In many of the hearts it is also possible to recognise a well-formed retro-aortic node. **Conclusions:** Despite the differences in the arrangement of the sinus node, mouse is an ideal model for further investigations of cardiac conduction, in particular to clarify the potential roles of the superior septal pathways and the retro-aortic node.

## 1. Introduction

The mouse is now widely used as a model for the study of cardiac diseases, both congenital and acquired. In this regard, it is generally accepted that, although there are subtle differences in anatomy when compared to the human heart, the differences are insufficiently great to question the value of using the animal as a suitable model. Thus, even when taking note of the subtle differences in anatomy, the interrelationships of the cardiac chambers and the ventricular outflow tract are sufficiently comparable to make the mouse an excellent model with regard to studies relating to the disposition and function of the conduction tissues. Little has been done thus far, however, to establish the specific arrangement of the components of the conduction tissues in the murine heart relative to the gross anatomical landmarks. An excellent account of the overall arrangement of the conduction tissues in the mouse had been provided by the end of the twentieth century [1]. We have recently compared the arrangement of the atrioventricular conduction axis in human and murine hearts [2] and also provided a review of the arrangement of the atrioventricular node in various species, including man and mouse [3]. In these studies, we emphasised that Lev and Thaemert had noted the presence, in the murine heart, of the paraspecific pathways as identified by Mahaim [4]. We further emphasised that the presence of these pathways has now achieved increased clinical significance [5]. This is because electrophysiological studies have shown that, in the murine heart, ventricular activation occurs from base to apex [6]. This contrasts with the apex-to-base patterns observed in the majority of mammalian hearts, including man [7]. This finding makes the murine heart even more important with regard to modelling of human disease, since we pointed out that, with knowledge of the site and extent of the superior septal pathways, experiments in the mouse could well resolve their function. Our earlier reviews, however, had been confined to discussions of the arrangement of the atrioventricular conduction axis and the atrioventricular node. There are other features of the conduction tissues that remain to be resolved, such as the function of the so-called “retro-aortic node” [8]. This component of the conduction tissues had been described in the murine heart [9], but Lev and Thaemert [1] had been unable to confirm this finding. With this in mind, therefore, and in the light of our neglect of the sinus node in our earlier study, along with the current interest in the substrates for interatrial conduction [10], we have extended our studies of the location of the conduction tissues in the mouse heart. In particular, we have provided further information on the relationships between all the histologically identifiable components of the conduction system and the gross anatomical landmarks.

## 2. Materials and Methods

We have re-examined the serial sections prepared from 8 hearts obtained from adult mice in which we previously compared the arrangement specifically of the atrioventricular conduction axis in murine and human hearts [2]. For the purposes of this study, we have prepared an additional 12 datasets, with 5 sectioned in the frontal plane, 3 in the sagittal plane, and 4 in the short axis of the ventricular mass. The study was approved by the Institutional Review Board of Extremadura University for the studies involving mice. (Code: No. 10/09/2022). All sections were retained, with one section stained using the trichrome technique from each five initially cut. When necessary, additional sections were mounted and stained from areas of particular interest. So as to provide details of the gross anatomy, again as in our previous investigation [2], we assessed datasets prepared using the technique of high-resolution episcopic microscopy [11]. The datasets had been prepared by Dr Tim Mohun whilst he was working at the Crick Institute in London. The datasets themselves are now housed in the archive of the Human Developmental Biology Resource and are available for direct study via the website of the archive (https://www.hdbr.org, accessed on 20 June 2023. For our continuing studies, we assessed datasets from the archive prepared from embryos sacrificed at embryonic day 18.5, along with neonatal mice sacrificed on the first, second, and third days of postnatal life. Each dataset, representing a three-dimensional reconstruction, could be sectioned in any desired plane. This permitted us to create figures so as to clarify the interrelations of the chambers pertinent to the location of the components of the conduction system and making comparisons with the landmarks used in the human heart [2,3].

## 3. Results

### 3.1. Gross Anatomy

**Figure 1:**
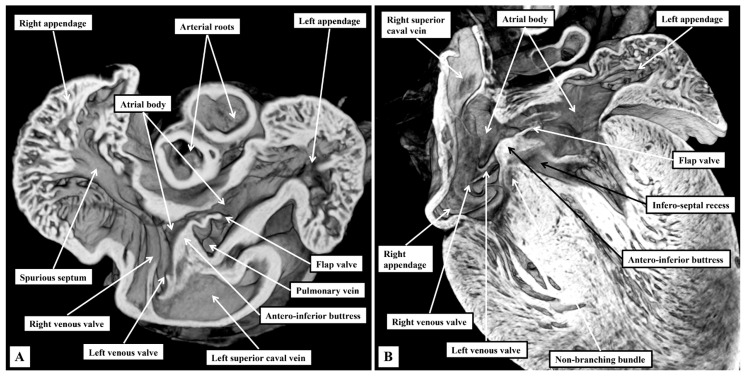
Details of gross atrial anatomy. The images, taken from three-dimensional datasets from a mouse on the first postnatal day, and prepared using high-resolution episcopic microscopy, show the features of the atrial chambers. (**A**) Shows a short-axis view looking from the apex towards the base of the heart. It contrasts the extent of the pectinate muscles within the atrial appendages. (**B**) Is a four-chamber cut. It shows well how the left venous valve buries itself in the myocardium of the antero-inferior buttress of the oval fossa having traversed between the orifices of the inferior caval vein and the left superior caval vein.

As we showed in our earlier study [2], despite the subtle differences, the basic arrangement of the cardiac chambers and the ventricular outflow tracts is such as to permit direct comparisons to be made with the features as observed in the human heart. In our previous study, however, we concentrated specifically on the arrangements of the atrioventricular junctions. We have now extended our observations so as to provide details of the location of all the components of the conduction system. In this regard, reflecting the difference in plantigrade as opposed to orthograde posture, there are differences at the level of the atrial chambers in the interrelations of the venous sinuses to the remainder of the components. So as to permit comparisons to be made with the anticipated human arrangements, we have purposely orientated our own figures, as far as possible, to reflect the situation as now seen in human hearts viewed in attitudinally appropriate fashion [12]. The atrial appendages, when viewed externally, are not as different in their shape when compared to the human arrangement. When account is taken of the extent of the pectinate muscles relative to the atrial vestibules, the differences between the morphologically right and left chambers are just marked as in the human heart (Figure 1A).

The pectinate muscles of the morphologically right appendage encircle the vestibule of the tricuspid valve, reaching dorsally to the orifice of the left superior caval vein (Figure 1A). On the left side, the pectinate muscles are confined within the morphologically left appendage, with the smooth vestibule of the mitral valve confluent dorsally with the smooth-walled atrial body (Figure 1B). Each atrial chamber possesses an appendage, a venous component, a vestibule, and retains part of the initial body derived from the primary heart tube. The cavities are separated by the septum. As in the human heart, the floor of the fossa is formed by the flap valve, derived from the primary atrial septum, which is anchored at the atrioventricular junction by a prominent antero-inferior buttress (Figure 1B).

**Figure 2:**
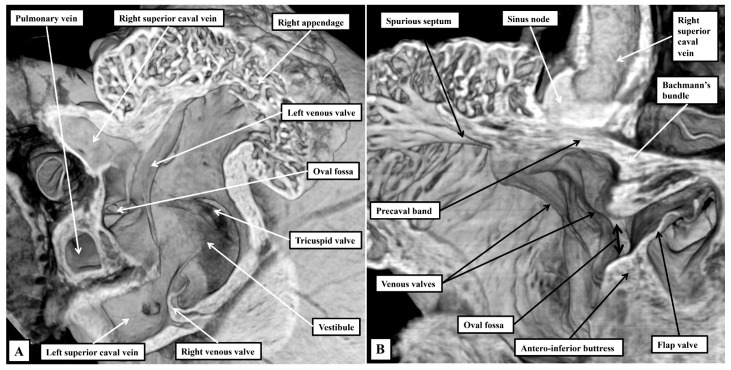
Details of the sinuatrial junctions in the right atrium. The images are again taken from a mouse sacrificed on the first postnatal day. (**A**) Is an oblique cut across the right atrium, showing the venous valves that form the boundaries of the sytemic venous sinus, and the solitary channel through which the pulmonary veins connect to the left atrium. (**B**) Is a “four chamber” cut, showing the site of the sinus node, and the continuation of the terminal crest through the precaval band to form Bachmann’s bundle.

The superior and dorsal rims of the oval fossa are infoldings of the atrial walls, with this feature particularly obvious dorsally. reflecting the entry of the pulmonary veins dorsally, rather than to the atrial roof as found in the human heart. Obvious and well-formed venous valves mark the boundary of the systemic venous sinus with the remainder of the right atrium, with the left venous valve being much better preserved than in the human heart (Figure 2A). The most obvious difference compared to the human heart is the presence in the murine heart of a left superior caval vein opening within the systemic venous sinus of the right atrium through the wide mouth of the coronary sinus (Figure 2A). As emphasised above, the pulmonary veins are also significantly different in the murine heart, opening dorsally through a common collecting channel which is adjacent to the atrioventricular junction. The terminal crest is less obvious in the right atrium when compared to its prominence in the human heart, but the spurious septum is much better formed (Figure 2B). The terminal groove is also a less obvious landmark in the murine heart, with potential significance for the different arrangement of the sinus node (see below). As in the human heart, nonetheless, the terminal crest passes in front of the orifice of the superior caval vein as the precaval band, which then continues as the anterior interatrial groove. Again as in the human heart, it is the continuation of the atrial walls through the precaval band and the anterior interatrial groove that constitutes Bachmann’s bundle [13] (Figure 2B).

**Figure 3:**
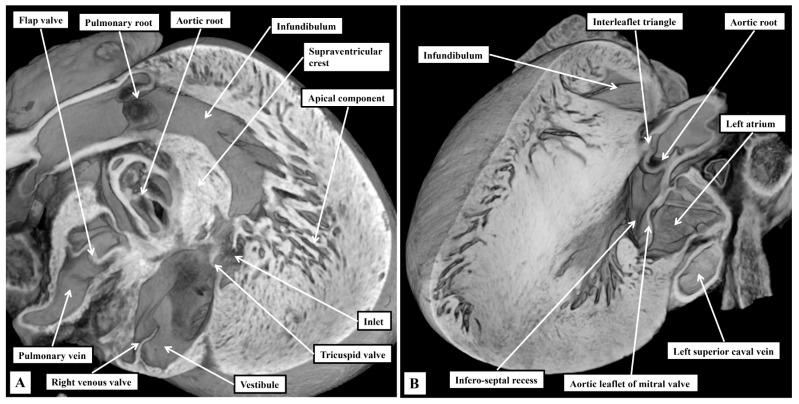
Details of ventricular morphology. The episcopic images, from a mouse sacrificed on the third postnatal day, show the salient features of the ventricular mass. (**A**) Shows an oblique cut through the right ventricle, showing its tripartite structure. (**B**) Is a similar oblique cut to show how, in the left ventricle, the aortic root is wedged between the mitral valve and the septum. Note the infero-septal recess of the outflow tract.

When assessing the ventricular chambers, the arrangements are comparable to the human situation in that each ventricle possesses an inlet, an apical component, and an outlet (Figure 3). The right ventricular outlet is well-formed infundibular sleeve (Figure 3A). In the outflow tract of the left ventricle, in contrast, the non-coronary and left coronary leaflets of the aortic valve are in fibrous continuity with one of the leaflets of the mitral valve. This permits the mitral valvar leaflet to be described as being aortic (Figure 3B). The other leaflet, supported by the parietal wall of the left ventricle, is well described as being mural. As we emphasised in our previous study [2], the left ventricular outflow tract has an extensive infero-septal recess. It extends dorsally between the ventricular septal surface and the aortic leaflet of the mitral valve to reach the parietal wall of the left ventricle at the crux. The recess is roofed by an area of fibrous continuity between the leaflets of the tricuspid and mitral valves (Figure 3B).

**Figure 4:**
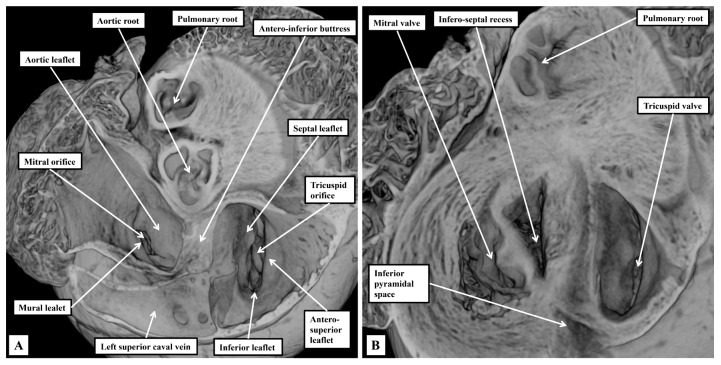
The relations of the inferior pyramidal space and the infero-septal recess of the left ventricular outflow tract. The episcopic sections are from a mouse sacrificed on the third postnatal day. In (**A**), the atrial walls have been cut back to the level of the atrial vestibules. Removal of the vestibules, as shown in (**B**), shows the location of the inferior pyramidal space, an extension of the inferior atrioventricular groove, relative to the infero-septal recess of the left ventricular outflow tract.

The right atrioventricular valve, although still described as being “tricuspid”, is frequently bifoliate rather than trifoliate (Figure 4A). In some hearts, it is possible to recognise a small inferior leaflet in addition to the better-formed septal and antero-superior leaflets. The mitral valve is obviously bifoliate, with the view of the ventricular short axis from the atrial aspect revealing the mural and aortic support provided to its leaflets, which close along a solitary zone of apposition. Dissection of the atrial walls shows how the diverging inferior vestibules of the atrioventricular valves form the walls of the superior extension of the inferior atrioventricular groove. This is the inferior pyramidal space (Figure 4B).

**Figure 5:**
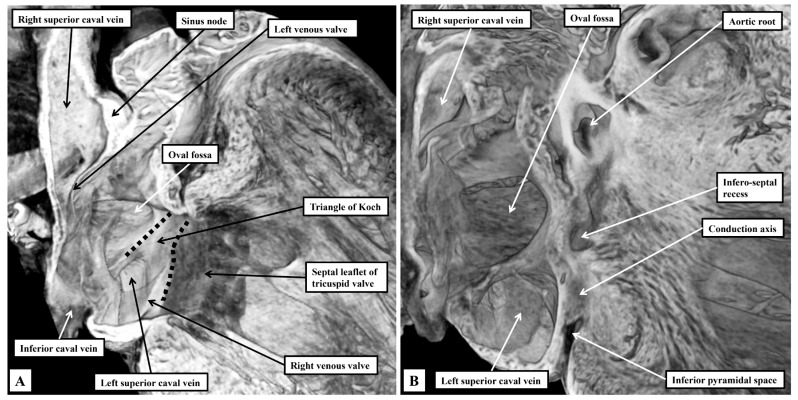
The triangle of Koch and the atrioventricular conduction axis. The images are taken from an episcopic dataset prepared from a mouse sacrificed on the first postnatal day. (**A**) Has been prepared to show the septal surface of the right atrium. Note that the right venous valve buries itself in the septal vestibule of the tricuspid valve. It is the commissure of the venous valves that enters the atrial walls between the oval fossa and the mouth of the coronary sinus that forms the posterior boundary of the triangle of Koch. The anterior boundary is the hinge of the septal leaflet of the tricuspid valve. Further dissection of the septal surface, as shown in (**B**), reveals how the apex of the inferior pyramidal space is directly adjacent to the infero-septal recess of the left ventricular outflow tract.

On the left side, the fibro-adipose tissues occupying the inferior pyramidal space separate the wall of the left superior caval vein from the underlying crest of the ventricular mass. As we showed in our previous reviews [2,3], it is the relationships between the inferior pyramidal space and the infero-septal recess that provide the landmarks for understanding the course of the atrioventricular conduction axis. As in the human heart, the inferior commissure of the venous valves, which initially separates the inferior extent of the right and left horns of the developing systemic venous sinus, enters the myocardium, separating the oval fossa from mouth of the coronary sinus, which in the human is the remnant of the left sinus horn. In the murine heart, because the left superior caval vein persists as the major channel draining the systemic venous return from the head and upper limbs, the mouth of the sinus is much larger than in the human heart (Figure 5).

The right venous valve in the murine heart buries itself in the atrial vestibule inferior to the orifice of the left caval vein (Figure 5A), a feature not obvious in the human heart. It is still possible, nonetheless, to recognise the triangle of Koch, with its apex pointing superiorly, and with the orifice of the left caval vein forming its base (Figure 5A). The atrial border is the tendon of Todaro, derived from the commissure of the fused venous valves separating the orifices of the right and left sinus horns. The ventricular border is formed by the hinge of the septal leaflet of the tricuspid valve (Figure 5A). Further dissections of the epicopic dataset then reveal the extent of the infero-septal recess of the left ventricular outflow tract, and its relation to the apex of the inferior pyramidal space (Figure 5B).

### 3.2. The Arrangement of the Conduction Tissues

**Figure 6:**
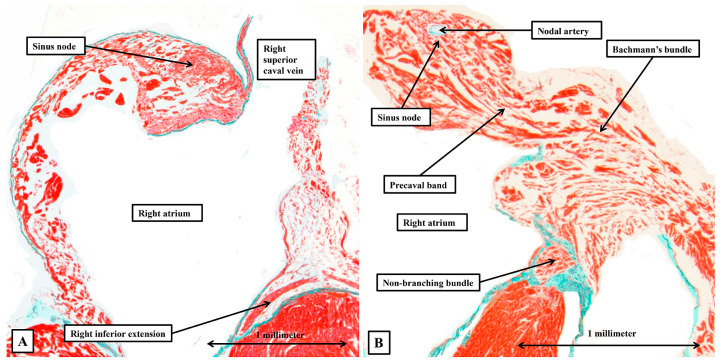
The sinus node and Bachmann’s bundle. The sections, shown in four-chamber fashion, reveal in (**A**) the location of the sinus node at the entrance of the superior caval vein to the right atrium. (**B**) Shows the transition from the sinus node, recognised in this section by its relationship to the nodal artery, to the working cardiomyocytes of the precaval band and Bachmann’s bundle. Masson’s trichrome stain technique.

As was well described by Lev and Thaemert [1], and as was shown by dissections of the episcopic datasets (Figure 2B and Figure 5A), the sinus node is a densely packed knot of cardiomyocytes draped across the ventral wall of the superior caval vein at its entrance to the morphologically right atrium (Figure 6).

When traced through serial sections, again as well described by Lev and Thaemert [1], the node extends into the terminal groove, with its cardiomyocytes becoming less densely packed, but arranged around a prominent nodal artery (Figure 6B). At its margins, the cells of the node transition to become working cardiomyocytes. The working cardiomyocytes are themselves then densely packed and well aligned as they aggregate together to form the terminal crest and Bachmann’s bundle, but then become more dispersed within the atrial walls (Figure 6B). It is also the working atrial cardiomyocytes that make up the walls separating the sinus and atrioventricular nodes (Figure 6).

**Figure 7:**
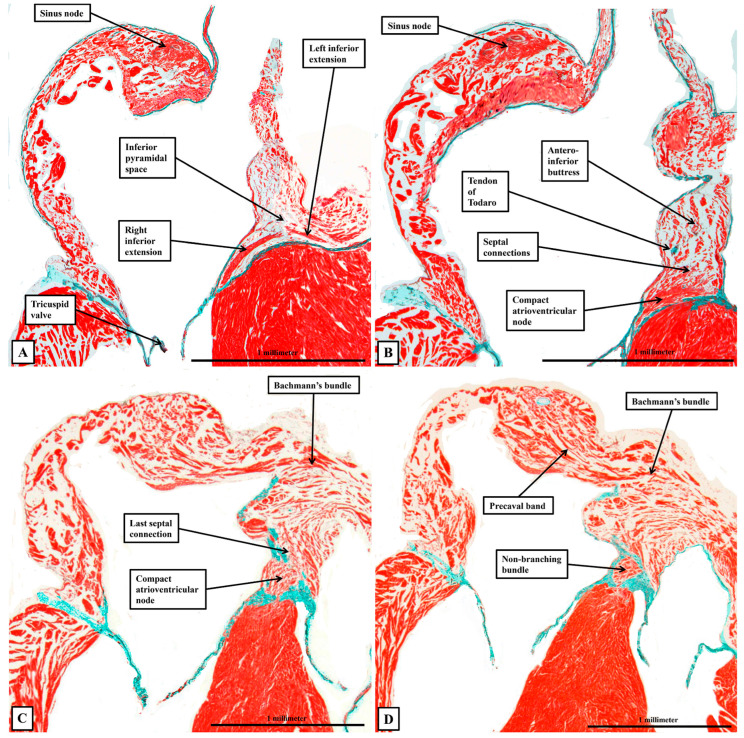
The atrial components of the conduction tissues. The images show a series of sections in the frontal plane taken from the murine heart and shown in “four chamber” projection. (**A**) Shows the rightward and leftward inferior extensions of the atrioventricular node, which occupy the vestibules of the tricuspid and mitral valvar orifices. (**B**) Shows how connections from the atrial septum fuse with the extensions to produce the compact atrioventricular node. (**C**) Shows the last septal connection before the axis becomes insulated as the non-branching bundle. In (**D**) shows the non-branching bundle surrounded by connective tissue.

With regard to the atrioventricular conduction axis, as might be expected, our current findings do no more than reinforce our recent accounts comparing the arrangement with the features found in the human heart [2,3]. For this study, however, we have prepared new datasets so as to be able to evaluate the findings using sections prepared in all three orthogonal planes. When assessing the sections prepared in the frontal plane, we were able to confirm that the compact component of the node is found within the antero-inferior buttress of the atrial septum, supported by the insulating tissues of the atrioventricular junction. Nodal cells can then be traced dorsally as extensions into vestibules of both the tricuspid and mitral valves (Figure 7A). The cardiomyocytes of the buttress of the oval fossa then join with the two inferior extensions to produce the compact component of the atrioventricular node (Figure 7B). As we showed in our earlier studies, in which we included images showing the arrangement in the human heart [2,3], the compact node is located toward the apex of the triangle of Koch, with the atrial border of the triangle formed by the tendon of Todaro, itself the extension of the commissure between the venous valves into the myocardial fold separating the orifices of the inferior and left superior caval veins (Figure 5A). At the apex of the triangle, the tendon fuses with an extension of the fibrous tissues supporting the septal hinges of the tricuspid and mitral valves. Immediately prior to the level of fusion, it is possible to recognise the last connection between the cardiomyocytes of the atrial septum and the atrioventricular node (Figure 7C). Once the axis has been insulated, it becomes the non-branching bundle (Figure 7D).

**Figure 8:**
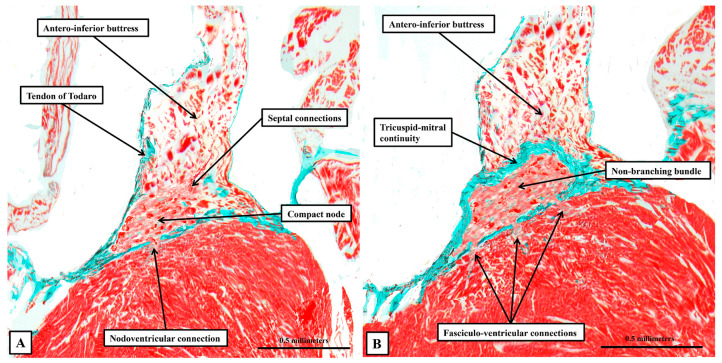
The transition from the atrioventricular node to the non-branching bundle. The images, from a different heart cut in the frontal plane, show how the insulation provided by fibrous continuity between the leaflets of the tricuspid and mitral valves (**B**) provides the means of distinguishing between the compact atrioventricular node (**A**) and the non-branching atrioventricular bundle (**B**). Note the presence, in both sections, of pathways producing myocardial continuity between the axis and the crest of the muscular septum. Note also that there is no difference in histology between the non-insulated and insulated components of the axis.

There is no obvious discernible difference in the histological make-up of the axis before or after its insulation (Figure 8).

**Figure 9:**
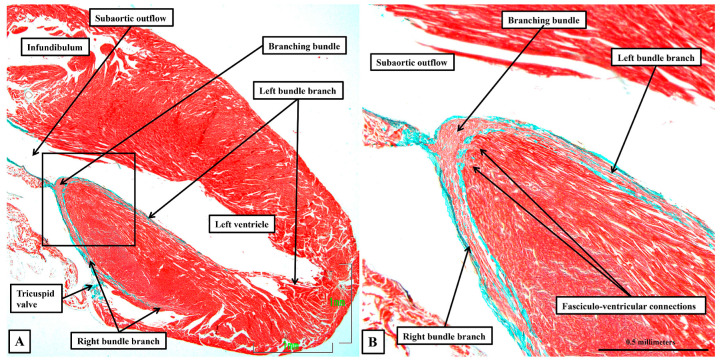
The branching atrioventricular bundle. The images, taken from a murine heart sections in the sagittal plane, show how the axis branches on the crest of the muscular ventricular septum (**A**), with the cardiomyocytes making up the right and left bundle branches ensheathed by fibrous tissue as they extend down the sub-endocardial surfaces of the septum. Note in (**B**) that the cardiomyocytes themselves are no larger than the working cardiomyocytes making up the ventricular septum.

The fibrous tissues insulating the axis from the atrial cardiomyocytes are part of the atrioventricular membranous septum, which forms part of the rightward wall of the infero-septal recess of the left ventricular outflow tract. The non-branching bundle itself extends along the ventricular septal crest within the recess, giving origin to its right and left branches at the entrance to the aortic root. Sections prepared by cutting the heart in its short axis confirm that the compact node, in addition to its inferior extensions, which provide inputs from the atrial vestibules, receives extensive direct connections from the working myocardium of the antero-inferior buttress of the atrial septum. When describing our earlier findings [2,3], we did not have access to any sections from hearts cut in their sagittal plane. Our new datasets prepared in this fashion add further information regarding the relationship between the overall conduction axis and the atrial and ventricular septal structures (Figure 9).

Having become insulated from the atrial myocardium to become the non-branching bundle, the axis then branches of the crest of the muscular ventricular septum to form the right and left bundle branches (Figure 9A). Both branches are ensheathed by fibrous tissue until the histologically specialised cardiomyocytes merge with working myocardium at the apexes of both right and left ventricles (Figure 9B), with the right bundle crossing the ventricular cavity through the prominent septoparietal trabeculation known as the moderator band. Although the axis and its right and left branches are wrapped within fibrous sheaths, however, there are multiple fenestrations within the components of the sheaths that separate the non-branching and proximal branching bundles from the crest of the muscular ventricular septum. These are the fasciculo-ventricular connections, or superior septal pathways (Figure 8B). In some of the hearts, there are additional direct connections between the compact atrioventricular node and the ventricular septum (Figure 8A). The specialised tissues of the axis pass through these fenestrations but then merge rapidly with the working myocardium of the ventricular septum (Figure 8). As was the case for the human heart, the cardiomyocytes making up the components of the conduction axis in the murine heart, including the right and left bundle branches and the superior septal pathways, are no larger than the adjacent working cardiomyocytes of the ventricular septum. It is a mistake to describe them as “Purkinje” cells. Of particular note, we were unable to use our gross histological sections to identify any cardiomyocytes within the ventricular septum itself that would bear comparison with the Purkinje cells as the latter entities are identified in ungulate hearts.

**Figure 10:**
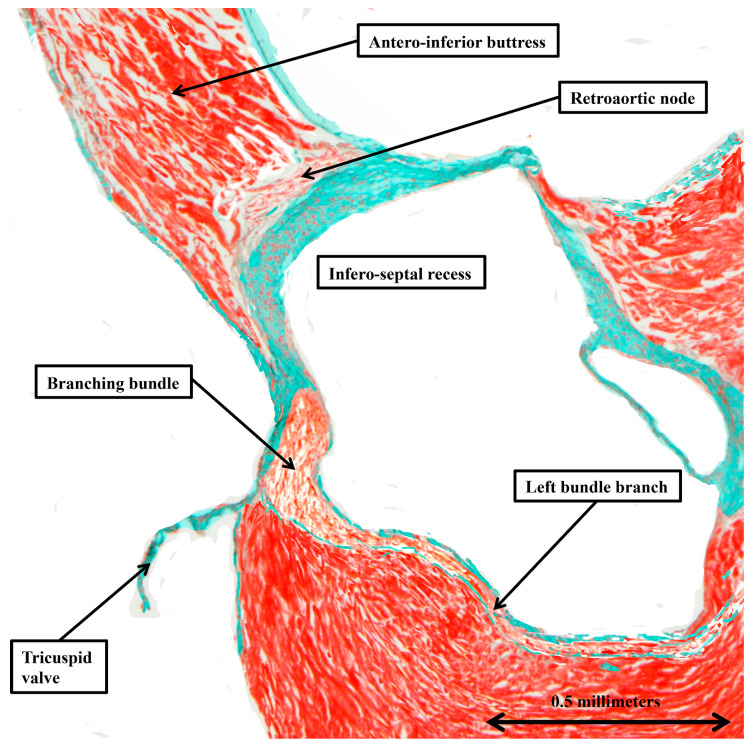
The retro-aortic node. The section, cut in the short axis of the ventricular mass, shows the location of the retro-aortic node at the base of the antero-inferior buttress of the atrial septum.

When the inferior extensions of the compact node are traced into the atrial vestibules, it can be seen that the rightward inferior extension is much more prominent than the leftward extension (Figure 7A). When assessing the sections stained using the trichrome technique, the extensions can be traced for limited distances before becoming indistinguishable from the working atrial myocardium. The rightward extension, nonetheless, is known to be part of the so-called primary ring, which can be shown in rat hearts, when using specific markers, to encircle the orifice of the tricuspid valve [8]. In the rat heart, it is then possible to show a second nodal structure present in the ventral atrial walls directly behind the aortic root. Evidence of this retro-aortic node is also present in the trichrome-stained sections from some, but not all, of the murine datasets (Figure 10).

## 4. Discussion

In this study, we have extended our investigation of the arrangement of the conduction tissues of the murine heart, as in our earlier studies making direct comparisons with the arrangements found in the human heart [2,3]. In the previous reviews, we discussed only the atrioventricular conduction axis and the atrioventricular node. Our ongoing investigations, in which we have addressed the entirety of the conduction tissues, along with the substrates for interatrial conduction, serve further to emphasise the potential for using the murine heart as a test bed for resolving ongoing issues regarding components that, thus far, have received scant attention. It is our hope that those with the capacity to examine the functional aspects of cardiac conduction will be stimulated by our account to provide the necessary experimental data. In this regard, in our earlier study devoted to the atrioventricular conduction axis [2], we had drawn attention to the potential role of the so-called “paraspecific” system for conduction initially identified by Mahaim [4]. Our current investigation, encompassing the entirety of the conduction system, now serves to emphasise the accuracy of the account provided quite some time ago by Lev and Thaemert [1]. Writing in 1973, these investigators had found previous accounts to be “fragmentary and incomplete”. As well as providing details of the well-recognised components of the conduction system, specifically the sinus node and the atrioventricular conduction axis, Lev and Thaemert had endorsed the presence of the superior septal pathways described by Mahaim as his paraspecific system [4]. Lev and Thaemert, however, had been unable to confirm a suggestion made by Nomura. According to the latter investigator, in addition to the regular atrioventricular node, there was a “ventral atrial system” [9]. Nomura had described this entity as a mass of specialised cardiomyocytes located at the intersection of the antero-inferior buttress of the atrial septum with the atrial vestibules. Although Lev and Thaemert were unable to identify this second node-like structure, we have been able to find evidence of its presence in our datasets, although it was not always as obvious as the obviously histologically discrete structure shown in Figure 10. It is the so-called retro-aortic node [8]. It is another structure with a function still to be confirmed, hopefully by those able to study cardiac conduction in the murine heart. In the human heart, it is likely to be involved in production of the arrhythmias that can be cured by ablations made from the non-coronary sinus of the aortic root [14].

Nomura had also suggested that extensions from the sinus node could be traced towards the atrioventricular node [9]. Lev and Thaemert had been unable to confirm this suggestion [1]. In this regard, our observations are in keeping with the conclusions drawn by Lev and Thaemert. The potential presence of pathways of specialised cardiomyocytes extending between the sinus and atrioventricular nodes had been a topic of significant controversy throughout the latter decades of the twentieth century. It is now accepted that no such insulated tracts are to be found in any way comparable to the ramifications of the ventricular conduction pathways [15]. It is the ordinary working myocardium that makes up the atrial walls between the nodes. The obvious aggregation of the working cardiomyocytes in the prominent muscle bundles such as the terminal crest or Bachmann’s bundle, nonetheless, serves to endorse the initial observation made by Bachmann himself when describing the bundle that now bears his name [13]. This topic has also become of significance recently, with suggestions made that there may be something “special” about the cardiomyocytes making up the bundle [10]. Our results in the mouse are in keeping with our findings in the human and swine hearts [16]. As we have suggested, it may well be that, when using techniques such as spatial transcriptomics, specific features may be found within the cardiomyocytes making up Bachmann’s bundle. Until that time, all that can be stated is that the atrial walls are made up of working cardiomyocytes.

Our findings also endorse the descriptions provided by Lev and Thaemert regarding the location of the sinus node [1]. The node can be well recognised in the episcopic datasets as a dense knot draped over the ventral wall of the right superior caval vein at its junction with the right atrium. The connections between the nodal cardiomyocytes and the atrial walls are much more dispersed. The node is not wedged within the terminal groove to the extent seen in the human heart. This likely reflects the difference in posture between man and mouse. The other major difference between man and mouse, with the left superior caval vein persisting as a regular venous channel in the mouse, influences the morphology of the triangle of Koch. The “triangle” is relative square in the murine heart, with the left venous valve also persisting to far greater extent compared to the arrangement in man. The membranous part of the septum, however, is much less well-developed than in the human heart.

With regard to the atrioventricular conduction axis, our further studies do no more than endorse our account in which we made direct comparisons with the arrangement as found in the human heart [2,3]. As we emphasised, it is the relationships of the infero-septal recess and the inferior pyramidal space which underscore the comparable arrangement of the atrioventricular conduction axis found in the human and murine hearts, with these features lacking in canine, porcine, and ungulate species [17]. It is the similarity of the atrioventricular conduction axis and the presence of multiple superior septal pathways which potentially make the mouse such a valuable model for investigation of the normal and abnormal pathways of the atrioventricular conduction. Much of the electrophysiological research on atrioventricular conduction has been carried out on species such as the rabbit or the dog. The fundamental differences in the architecture of the atrioventricular node and the non-branching bundle in these species when compared to the human arrangement have rarely been recognised by those making physiological experiments. The arrangement in the murine heart, with inferior extensions joining together with atrial septal contributions to form a compact atrioventricular node, is directly comparable with the human architecture. As in the human heart, furthermore, the conduction axis becomes insulated from the atrial myocardium by an area of fibrous tissue that produces continuity between the leaflets of the tricuspid and mitral valves. In both human and murine hearts, there is little, if any, difference in the histological arrangement of the axis as it transitions to become the non-branching atrioventricular bundle, with no obvious formation of a “lower nodal bundle” as suggested by some investigators for the human heart [18].

Despite the potential significance of further studies related to the retro-aortic node, it is the presence of the extensive fasciculo-ventricular connections that is the most significant feature of the murine conduction system. As we have emphasised, it was these pathways that Mahaim suggested provided a “paraspecific” system for atrioventricular conduction [4]. Subsequent to Mahaim’s account, the pathways have received scant recognition, although they were recognised by Hecht and his colleagues when they reviewed the pathways for atrioventricular conduction [19]. As we have also emphasised, the presence of the pathways in the murine heart was recognised by Lev and Thaemert [1], although they did not illustrate them. We have found the pathways to be extensive, although once having perforated the fibrous insulating tissues they merge rapidly with the working myocardium of the crest of the muscular ventricular septum. The pathways were recognised by the group of electrophysiologists working in Utrecht as the substrates for the base-to-apex ventricular activation they had identified in the murine heart [6]. Little attention has been directed to the potential role of these pathways in ventricular activation in the human heart, particularly in the setting of so-called “selective pacing of the left bundle branch” [20]. The presence of the pathways has also been ignored by those who have recently identified cardiomyocytes within the substance of the ventricular septum on the basis of their reaction to an antibody raised against the MYL4 gene [21]. Those identifying the cardiomyocytes describe them as “Purkinje cells”. This creates problems in itself, since the cardiomyocytes identified by Purkinje are to be found only in the hearts of ungulates. In human and murine hearts, at least on the basis of gross histological studies, there is no evidence to show that such cardiomyocytes are part of the ventricular conduction system. It is surely the case that further experimental studies using the murine heart can clarify their significance, as is also the case for the retro-aortic node. The analogue of the retro-aortic variant is known to function, in most individuals, as the connecting node in the setting of congenitally corrected transposition. As was shown by a recent study, it is the ablation from the pulmonary root that is able to cure atrial tachycardias in this setting [22]. Our current findings suggest that its potential role in normal conduction can be elucidated by appropriate investigations in the murine heart. We recognise that our own investigation has its limitations. It is also a direct extension of our previous accounts, in which we made direct comparisons with the arrangements of the atrioventricular conduction axis and atrioventricular node as found in the human heart [2,3]. In all our studies, we have studied the disposition of the conduction axis using only standard histological stains. In an elegant investigation using electron microscopy, an intensive contribution of neural elements was revealed in the murine atrioventricular node [23]. Further studies are justified to extend this observation, and to establish whether the ventricular pathways are similarly richly innervated.

## 5. Conclusions

Despite the differences in the arrangement of the sinus node, the most important feature of the mouse heart is the presence of extensive fasciculo-ventricular connections. As we have emphasised, it was these pathways that Mahaim suggested provided a “paraspecific” system for atrioventricular conduction [4]. The pathways were recognised by the group of electrophysiologists working in Utrecht as the substrates for the base-to-apex ventricular activation they had identified in the mouse heart [6]. It is surely the case that further experimental studies using the mouse heart can clarify their role and also elucidate any role of the retroaortic node in normal or abnormal atrioventricular conduction.

## Data Availability

The data that support our research results can be found in the references cited in the manuscript.
